# Haplotype Analysis of PPARγ Gene Polymorphisms and the Lipoprotein (a) Level

**Published:** 2018-07

**Authors:** Chao SHEN, Wei FAN, Hui-Jian XIE, Ming WU, Zheng-Yuan ZHOU, Zhi-Rong GUO, Chen DONG

**Affiliations:** 1. Dept. of Epidemiology, School of Public Health, Medical College of Soochow University, Suzhou, China; 2. The Center for Disease Control and Prevention of Suzhou Industry Park, SuZhou, China; 3. Center for Disease Control, Nanjing, Jiangsu Province, China; 4. Center for Disease Control of Changshu, Suzhou, China

**Keywords:** Peroxisome proliferator-activated receptor γ, Lipoprotein (a), Haplotype, Polymorphism

## Abstract

**Background:**

Lipoprotein (a) [Lp(a)], as an independent risk factor for cardiovascular disease, is more likely to be genetically determined according to the increasing evidence of epidemiologic and clinical studies in recent years. Peroxisome proliferator-activated receptor (PPAR) γ, the ligand-activated transcription factors, was considered as an indispensable role in the process of lipid metabolism. This study was designed to explore the associations of three single-nucleotide polymorphisms (SNPs) and the haplotypes of the peroxisome proliferator-activated receptor (PPAR)γ gene with the level of Lp(a).

**Methods:**

Participants were recruited under the framework of the PMMJS (The Prevention of Metabolic Syndrome (MS) and Multi-metabolic Disorders in Jiangsu Province of China Study) from Apr 1999 to Jun 2004. Overall, 644 subjects were randomly selected and 3 SNPs of PPARγ gene (rs10865710, rs1805192, rs4684847) were genotyped.

**Results:**

After adjusting for age, sex, cigarette smoking, alcohol drinking, waist circumference and body mass index, rs4684847 was significantly associated with Lp (a). The presence of the rs4684847 T allele (CT+TT) have a lower level of Lp (a) than the allele (CC) in the dominant model, mean difference was −27.30 (95% *CI*: −52.88∼−1.73) mg/L, *P*<0.05. G-P-T and G-A-T haplotype were associated with lower levels of Lp (a) (*P*=0.0041 and <0.0001), mean difference was 49.79 (95% *CI:* −97.52∼−2.06) mg/L and 17.75 (95% *CI*: −25.75∼−9.75) mg/L.

**Conclusion:**

PPAR gamma polymorphisms (rs10865710, rs1805192, rs4684847) and haplotypes may be the genetic risk factors for Lp (a) level.

## Introduction

Lipoprotein (a) [Lp(a)] is a circulating lipoprotein consisted of apolipoprotein B_100_ and apolipoprotein(a) linked by a disulfide bond, which is similar in lipid composition to apoB of low-density lipoprotein (LDL-C). Lp(a) is not only bound up with coronary heart disease (CHD) and stroke but also an independent risk factor for cardiovascular disease(1, 2). Levels of Lp(a) in individuals are highly stable for years and are weakly correlated with the well-known risk factors for CHD, such as smoking status, blood pressure(BP), total cholesterol (TC), triglycerides (TG), body mass index (BMI), and diabetes mellitus, suggesting that the level of Lp(a) in humans is probably genetically determined (3). Number of evidence has supported the assumption. The concentration of plasma Lp(a) was negatively correlated with numbers of KIV-2 (lipoprotein(a) kringle IV type 2) repeats, and people with KIV-2 repeats had increased risk for myocardial infarction(MI)(4). SNPs located at 5′-end of apolipoprotein (a) (LPA) gene were significantly associated with plasma Lp(a) concentration in South Asians, Chinese, and European Caucasians (5).

Peroxisome-activated receptors (PPARs) are ligand-activated transcription factors belonging to the nuclear receptor superfamily. Since all of PPAR isoforms (PPARα, PPAR δ and PPAR γ) were involved in regulating adipocyte differentiation, lipid storage, glucose metabolism, and the expression of related genes, polymorphisms of PPAR could also possibly have some effect on the level of Lp(a). The relationship between PPARα/γ polymorphisms gene-gene interaction and haplotype of PPARα with Lp(a) level has been proved in our previous researches (6,7). However, whether the haplotype of PPARγ associated with Lp(a) level is still unknown. Consequently, haplotype was established using the three single-nucleotide polymorphisms (SNPs) (rs10865710, rs1805192, rs4684847) to test the correlation.

## Materials and Methods

### Subjects

In this cross-sectional study, the participants were recruited under the framework of the PMMJS initiated from Apr 1999 to Jun 2004 (8). PMMJS aimed to estimate the MS prevalence in Jiangsu Province, China and 6400 persons aged 35–74 yr old were selected from this Province in baseline survey based on the economic condition of sample site by multi-stage sampling.4083 participants (89.11%) received follow-up examination include age, sex, smoking status, alcohol intake, family disease history and metabolic variables. After excluding subjects experienced stroke or cardiovascular disease (n=36), type 2 diabetes (n=289), missing data (n=133) or body mass index (BMI) <18.5 kg/m^2^ (n=27), there were 3598 subjects (9). Overall, 644 subjects were randomly selected from the remaining. Subjects selected were similar to those not selected in terms of age, sex, smoking, alcohol, family disease history and metabolic variable. Blood samples were collected at baseline from the 644 subjects and analyzed for genotype.

All the participants signed the informed consent form. The study was approved by the Ethics Committee of Soochow University.

Table 1 displays the baseline characteristics of the studied population.

**Table 1:** Baseline characteristics of the studied samples with sex

***Variables***	***Total(n=644)***	***Male(n=234)***	***Female(n=410)***	P-values*
Age (yr)	50.52±9.39	51.10±9.91	50.20±9.08	0.241
Smoking (n, %)	163(25.3)	148(63.2)	15(3.7)	<0.001
Alcohol (n, %)	117(27.5)	147(62.8)	30(7.3)	<0.001
WC (cm)	81.17±10.26	83.65±10.10	79.95±10.12	<0.001
BMI (kg/m^2^)	23.01±3.14	23.07±3.00	22.98±3.23	0.733
FPG (mmol/L)	5.04±0.73	5.01±0.77	5.05±0.71	0.525
Lp(a) (mg/L)	134.68(68.60–223.75)	135.19(63.25–225.50)	131.50(74.79–218.25)	0.899

Note: means±standard deviation for age, WC, BMI, and FPG; median and interquartile range for Lp(a)

*: *P-*values less than 0.05 were considered statistically significant

### Body measurements and laboratory methods

A standard questionnaire was administered by trained staff to collect demographics and lifestyle risk factors data from participants. Body weight, height, and waist circumference (WC) were measured according to standardized procedures, and BMI was calculated as weight in kilograms divided by the square of the height in meters. Blood samples were collected in the morning after at least 8 h of fasting. All plasma and serum samples were frozen at −80 °C until laboratory testing. Concentrations of Lp(a) were detected by ELISA. Fasting plasma glucose (FPG) was measured using an oxidase enzymatic method. The concentrations of high-density lipoprotein cholesterol (HDL-C) and triglycerides (TG) were assessed enzymatically in an automatic biochemistry analyzer (Hitachi, Japan). All laboratory analyses were performed at the same laboratory. The method of investigation during follow-up was the same as that used at baseline.

### SNP and haplotypes selection, genomic DNA extraction, and genotyping

We selected three SNPs within PPAR γ gene using the following methods: 1) minor allele frequency (MAF) >0.05; 2) SNPs of PPAR γ gene has ever been reported related to lipid metabolism abnormalities; 3) Selected SNPs in functional areas of the gene fragments, or in the region which may change the function; 4) SNPs proved to have gene-gene interaction with Lp(a) (6). Three SNPs (rs10865710, rs1805192, rs4684847) were recruited and eight haplotypes(C-P-C, G-P-C, C-A-C, C-P-T, G-A-C, G-P-T, C-A-T, G-A-T) will be analyzed in this study. Genomic DNA from participants was extracted from ethylenediaminetetraacetic acid-treated whole blood, using the DNA Blood Mini Kit (Qiagen, Hilden, Germany) according to the manufacturer’s instructions. The frequent and minor alleles for the three SNPs (rs10865710, rs1805192, rs4684847) were detected by TaqMan fluorescence probe. ABI Prism 7000 software and allelic discrimination procedure were used for genotyping of forementioned three SNPs. The 25 μl reaction mixture included 1.25 μl SNP genotyping assays (20×), 12.5μl Genotyping Master Mix (2×), and 20 ng DNA. The conditions were as follows: initial denaturation for 10 min at 95 °C, denaturation for 15 sec at 92 °C, an annealing and extension for 90 sec at 60 °C, for 50 cycles.

### Statistical analysis

Continuous variables of participants were calculated as means with standard deviation or median with interquartile range according to their distribution, and the categorical variables were presented as percentage. Chi-square test (χ^2^) was used to examine differences in the categorical data distribution. Further, continuous variables were analyzed using two-sample t-test or ANOVA for comparison between groups. A value of *P*<0.05, using two-sided tests, was considered statistically significant. For the purpose of quality control, deviation from the Hardy-Weinberg equilibrium (HWE) was used to detect genotype typing errors by Fisher’s exact test. Linkage disequilibrium (LD) between polymorphisms was estimated by using SHEsis (http://analysis.bio-x.cn). The association of SNP polymorphisms and haplotypes with Lp(a), as well as the mean difference and corresponding 95% confidence interval (CI) were all calculated using SNPstats software in a generalized linear model (http://bioinfo.iconcologia.net/SNPstats).

## Results

Overall, 644 participants (234 males and 410 females) were recruited in this study. The percentage of cigarette smoking, alcohol drinking, and WC were higher in males than in the females (*P*<0.05). We did not find any difference in age, BMI, FPG and Lp(a) between males and females. The associations between PPARγ polymorphisms and Lp(a) are shown in Table 2. After adjusting for age, sex, cigarette smoking, alcohol drinking, waist circumference and body mass index, the presence of the rs4684847 T allele(CT+TT) had a lower level of Lp(a) than the allele(CC) in the dominant model (Mean difference: −27.30; 95% *CI*: −52.88∼−1.73 mg/L, *P <* 0.05).

**Table 2:** Genotype for the 3 SNPs in PPARγ gene according to Lp(a) level

***Genotype***	***Frequency(%)***	Mean Difference**	P-values*
rs10865710			
CC	292(45.3)	3.07(−21.79∼27.93)	0.81
CG+GG	352(54.7)		
C	873(67.8)		
G	415(32.2)		
rs1805192			
PP	327(50.8)	−16.03(−40.69∼8.62)	0.2
PA+AA	317(49.2)		
P	904(70.2)		
A	384(29.8)		
rs4684847			
CC	413(64.1)	−27.30(−52.88∼−1.73)	0.037
CC+TT	231(35.9)		
C	1026(79.7)		
T	262(20.3)		

**: Adjusted for age, sex, cigarette smoking, alcohol drinking, WC, and BMI.

*: *P-*values less than 0.05 were considered statistically significant

However, the genotypes of rs10865710 and rs1805192 were not associated with Lp(a) level. Among 8 haplotypes, two haplotypes (G-P-T and G-A-T) were significantly associated with a lower level of Lp(a) (G-P-T, Mean Difference: −49.41 (−97.21 ∼ −1.62), *P*=0.0041 ; G-A-T, Mean Difference: −17.59 (−25.39 ∼ −9.79), *P*<0.0001) comparison with the most common haplotype of C-P-C (Table 3).

**Table 3:** Haplotypes of PPARγ gene with Lp(a) level

***Haplotype***	***Rs10865710***	***Rs1805192***	***Rs4684847***	***Frequency***	Mean difference (95%ci)**	P-value*
1	C	P	C	0.3733	0	
2	G	P	C	0.1754	26.95 (−2.19 ∼ 56.09)	0.07
3	C	A	C	0.1651	−0.32 (−27.78 ∼ 27.14)	0.98
4	C	P	T	0.1059	−2.19 (−35.94 ∼ 31.56)	0.9
5	G	A	C	0.0828	−2.95 (−38.96 ∼ 33.06)	0.87
6	G	P	T	0.0473	−49.41 (−97.21 ∼ −1.62)	0.043
7	C	A	T	0.0335	−36.04 (−99.51 ∼ 27.43)	0.27
8	G	A	T	0.0167	−17.59 (−25.39 ∼ −9.79)	<0.0001

**: Adjusted for age, sex, cigarette smoking, alcohol drinking, WC, and BMI.

*: *P-*values less than 0.05 were considered statistically significant

## Discussion

In the present study, we aimed to investigate the association between PPARγ gene and Lp(a) focusing on its polymorphism and haplotypes in a Chinese Han population. The haplotypes G-P-T and G-A-T, as well as rs4684847, were associated with Lp(a) level after adjusted for age, sex, cigarette smoking, alcohol drinking, waist circumference and body mass index.

PPARγ gene is highly expressed in adipose tissue and plays an important role in the regulation of adipocyte differentiation, lipid metabolism, and storage by up-regulating the transcriptional activity of the aP2(adipocyte P2), lipoprotein lipase, and phosphoenolpyruvate carboxykinase gene promoters (10, 11). The physiological function of lipoprotein lipase was considered to decompose triglyceride(TG) which is the core component of lipoprotein, suggest that PPARγ may reduce the concentration of lipoprotein. By addressing Pro12Ala polymorphism of PPAR-γ2, Ala/Ala homozygotes had a reduction in serum triglyceride compared to Pro/Pro carriers (12). There was a significant relationship between blood pressure, BMI, and the rs4684847 polymorphism. The T allele carriers have approximately 40% reduction in the risk of premature cardiovascular death compared to the CC genotype (13).

Many complex diseases in human beings could be influenced by multiple interactive genes. Haplotype analysis is generally used to explore the association between the diseases and multiple genes by testing some tag SNPs so as to reduce the workload of researches and provide more powerful and accurate results concomitantly. Haplotypes will generally be useful in refining SNP–phenotype associations if there are significant interactions among the SNPs in their effect on the trait even when LD is low (14). Since a significant gene-gene interaction among the three SNPs has been detected in previous study, we perform this study in order to further refine the association between these polymorphisms and Lp(a).

Apart from the statistical significance, we also concerned about the biological mechanism underlying these haplotypes. Lp(a), which can pass through the endothelial barrier and preferentially retain in the arterial intima by binding to the extracellular matrix via both apoB and apo(a), is the primary carrier of oxidized phospholipids associated with vascular inflammation and atherosclerosis. It also proved to be primary regulators in smooth muscle cell proliferation, and plaque inflammation and instability which are all key processes in atherosclerosis in vitro and animal studies (15). Lp(a) is an important risk factor for cardiovascular disease. Haplotypes of PPARs were related to cardiovascular disease risk factors including C-relactive protein, abnormal body weight and essential hypertension (16, 17).

To investigate the link between the two, we did this research. The liver is the major organ accounting for the clearance of Lp(a) and lipid metabolism. Effect of troglitazone (PPARγ agonist) improve lipid metabolism are mediated by PPARγ gene in liver cells in mice (18, 19), but the mechanism is still unknown. We suppose PPARγ gene regulates Lp(a) level by the way of affecting transcriptional activity of its downstream gene. “The Pro-to-Al exchange represents a genetic marker for the functional mutation that could reside in the promoter region of the PPARγ gene and result in reduced expression of PPARγ protein” (20). Moreover, all these lead to the suppression of free fatty acid release from fat tissue (20). The plasma level of Lp(a) cannot be decreased by statins or other lipid-lowering drugs except niacin, but the long-term efficacy and safety of niacin are still unclear. It is important for us to explore another pathway to lower plasma level of Lp(a) to prevent and treat cardiovascular disease. The dual PPARα/γ agonist aleglitazar increase adiponectin, reduce hepatic fat accumulation, augment circulating angiogenic cell migration and enhance neoangiogenesis, thus markedly reduce formation of atherosclerotic plaques and prevent the happening of the cardiovascular disease in mice experiments (21). Its potential application prospect was viewed although has not been confirmed in human beings.

Limitations should be considered. Firstly, the concentrations of Lp(a) vary significantly among different races. Blacks have the highest Lp(a) levels among all populations, followed by Indians and white (22). Because our study was limited to Chinese Han population only, studies involving larger and multi-racial samples still need to be performed to further verify our results. Secondly, we just examine 3 SNPs of PPARγ. The result we summarized could be one-sided or even wrong. Then recruit more SNPs is also necessary.

## Conclusion

We tested the association between PPARγ gene polymorphisms (rs10865710, rs1805192, rs4684847) and Lp(a) levels using both single-locus and haplotype analyses. Our results may support some previous findings that have been observed in various studies. As the level of Lp(a) may be involved with a complex genetic basis, relevant biological experiments require to be conducted to further unravel the exact mechanisms underlying the observations.

## Ethical considerations

Ethical issues (Including plagiarism, Informed Consent, misconduct, data fabrication and/or falsification, double publication and/or submission, redundancy, etc.) have been completely observed by the authors.
